# Novel Approach for Coexpression Analysis of E2F1–3 and MYC Target Genes in Chronic Myelogenous Leukemia

**DOI:** 10.1155/2014/439840

**Published:** 2014-08-10

**Authors:** Fengfeng Wang, Lawrence W. C. Chan, William C. S. Cho, Petrus Tang, Jun Yu, Chi-Ren Shyu, Nancy B. Y. Tsui, S. C. Cesar Wong, Parco M. Siu, S. P. Yip, Benjamin Y. M. Yung

**Affiliations:** ^1^Department of Health Technology and Informatics, Hong Kong Polytechnic University, Lee Shau Kee Building, Hung Hom, Kowloon, Hong Kong; ^2^Department of Clinical Oncology, Queen Elizabeth Hospital, 30 Gascoigne Road, Kowloon, Hong Kong; ^3^Bioinformatics Center, Chang Gung University, Taoyuan 333, Taiwan; ^4^Beijing Institute of Genomics, Chinese Academy of Sciences, Chaoyang District, Beijing 100029, China; ^5^Department of Computer Science, Informatics Institute, University of Missouri, Columbia, MO 65211, USA

## Abstract

*Background*. Chronic myelogenous leukemia (CML) is characterized by tremendous amount of immature myeloid cells in the blood circulation. E2F1–3 and MYC are important transcription factors that form positive feedback loops by reciprocal regulation in their own transcription processes. Since genes regulated by E2F1–3 or MYC are related to cell proliferation and apoptosis, we wonder if there exists difference in the coexpression patterns of genes regulated concurrently by E2F1–3 and MYC between the normal and the CML states. *Results*. We proposed a method to explore the difference in the coexpression patterns of those candidate target genes between the normal and the CML groups. A disease-specific cutoff point for coexpression levels that classified the coexpressed gene pairs into strong and weak coexpression classes was identified. Our developed method effectively identified the coexpression pattern differences from the overall structure. Moreover, we found that genes related to the cell adhesion and angiogenesis properties were more likely to be coexpressed in the normal group when compared to the CML group. *Conclusion*. Our findings may be helpful in exploring the underlying mechanisms of CML and provide useful information in cancer treatment.

## 1. Introduction

Chronic myelogenous leukemia (CML) is a clonal myeloproliferative disorder that is characterized by the premature circulation of many immature myeloid cells in the blood stream [1]. The incidence rate of CML is about 1-2 per 100,000 per year. CML accounts for 20% of all leukemias affecting adults with a median age of 45 to 55 years [2]. The characteristics of CML at the cellular level include increased proliferation, increased resistance to apoptosis, and alterations in adhesion properties of leukemic progenitors [1]. Recently, there are many more studies on the analysis of microarray gene expression profiles in CML. Most of them investigate the function of differentially expressed genes such as the study to explore the relationship between pathways and differentially expressed genes from untreated CML patients in the chronic phase [3]. However, few studies are available on the coexpression analysis.

Transcription factor (TF), a kind of transacting factor, plays the most vital role in the regulation of gene expression and process of signal transduction [4]. E2F family of transcription factors is important to control cellular proliferation by regulating transcription of various genes involved in DNA replication, DNA repair, mitosis, and cell cycle progression [5]. According to structure-function studies and amino acid sequence analysis, members of the E2F family can be classified into two main subclasses: activators E2F1–3 and repressors E2F4–8 [5]. The transcription activators E2F1, 2, and 3 are vital for cell cycle progression, especially in the G1/S transition process [6]. The protooncogene c-myc encodes a transcription factor (MYC) that can induce both cell proliferation and apoptosis [7]. As a transcription factor, MYC can both activate and repress transcription of target genes. High-throughput techniques have shown that MYC-activated genes are involved in growth, protein synthesis, and mitochondrial function. Most of MYC-repressed genes participate in the interaction and communication between cells and their external environment, and several genes are found to have antiproliferative or antimetastatic properties [8]. In addition, E2F1–3 and MYC are reciprocally regulated in the transcription process to form positive feedback loops among them [9].

Target genes regulated by the same TF tend to be coexpressed, and the coexpression degree is increased if genes share more TFs [10]. Moreover, coexpression analysis has been used to study functionally related genes since the coexpressed genes are more likely to participate in the similar cellular processes and pathways [11]. Furthermore, coexpressed genes are different in different states and cell types [12]. As a result, coexpression pattern analysis is a powerful strategy for grouping genes and further analyzing the underlying mechanisms of diseases. The different coexpression pattern can be regarded as the signature of a disease.

Since target genes regulated by E2F1–3 or MYC are related to cell proliferation and apoptosis, we wonder if there exists difference in the coexpression patterns of genes regulated concurrently by E2F1–3 and MYC between the normal and the CML states. In order to answer this research question, we proposed a method to explore the difference in the coexpression patterns by identifying a disease-specific cutoff point for coexpression levels that classified the coexpressed gene pairs into strong and weak coexpression classes so that the class was best coherent with the disease phenotype. Traditional methods on the coexpression analysis identify significantly coexpressed gene pairs by calculating a *P* value of correlation coefficient for each gene pair individually, which cannot reflect the overall difference between two different groups. Our method calculated all the correlation coefficients in each group to form two different cumulative distributions including all the gene pairs, which can identify the difference between two different groups from the overall structure. Also, the different coexpression pattern reflected the biological alterations in CML compared to the normal state. Annotation of the candidate target genes and mapping the coexpressed gene pairs to the annotated gene pairs from enriched process networks provided important information to understand the underlying mechanisms of the CML and the normal states.

## 2. Methods

### 2.1. Microarray Expression Data

Microarray technology is used to monitor the expression levels of thousands of genes in cells simultaneously [13]. Gene expression analysis across different conditions, the normal and the disease states, may contribute much to the exploration of disease mechanisms. In this study, we analyzed the microarray dataset GSE5550, normalized by variance stabilizing transformations (VSN) method, which is publicly available on the* Gene Expression Omnibus* (*GEO*) repository [3]. The data were obtained from gene expression measurements of 8,537 unique mRNAs. CD34+ hematopoietic stem and progenitor cells were collected from the bone marrow of patients with untreated CML in the chronic phase and health controls [3]. The subjects recruited for this dataset are Caucasians in Germany. The CML group consisted of nine patient samples, and the control group included eight normal samples. In this dataset, a gene may be interrogated by more than one probe. In this case, we took the average of all the probes for the same mRNA [14, 15].

### 2.2. Identification of Candidate Target Genes Regulated Concurrently by E2F1–3 and MYC

The interactions between TFs (E2F1, E2F2, E2F3, and MYC) and target genes (TGs) were obtained from* prediction of transcriptional regulatory modules *(*PReMod*) database [16]. TF binding sites are often clustered together, called cis-regulatory modules (CRMs).* PReMod* database predicts relationships between TFs and their TGs based on the binding affinity and conservation of CRM. It consists of more than 100,000 computationally predicted modules within the human genome [16]. These modules give a description of 229 potential transcription factor families and are the first genome-wide collection of predicted regulatory modules for the human genome [17]. In this study, we called the set of TF binding predictions (TF-TG pairs) from* PReMod* a molecular interaction set. This set was regarded as the reference data. After obtaining the TGs of each TF (E2F1, E2F2, E2F3, and MYC) individually, we identified the common TGs of these four TFs, which were regarded as the candidate target genes for further analysis. The flowchart is shown in Figure S1 Supplementary Material available online at http://dx.doi.org/10.1155/2014/439840.

### 2.3. Coexpression Measure

We chose Pearson correlation coefficient as the similarity measure. It is represented by the direction cosine between two vectors normalized by the subtraction of their own means, and its value accounts for the angle between two feature vectors instead of the vector lengths. Moreover, Pearson correlation coefficient numerically indicates the biological relationship of two genes but does not vary with the magnitudes of their expression profiles [11, 18]. In general, similarity measure is a kernel function between two feature vectors. In this study, each feature vector consisted of the expression intensity of a gene across all the samples in the normal group or the CML group, respectively. The correlation coefficient of any two genes among the candidate target genes was calculated. We took the absolute value of correlation coefficient (|*r*|) since the coexpression measure output a scalar in the range from 0 to 1 where a high output indicated a strong biological relationship in either positive or negative direction, and a low output indicated a weak biological relationship. The coexpression level was denoted by *C*_*d*_(*i*, *j*) if two expression profiles were extracted from samples of the disease (CML) group and *C*_*n*_(*i*, *j*) for the normal group, shown in Formulas (1) as follows:

(1) Cd(i,j)=|cor(xdi,xdj)|,Cn(i,j)=|cor(xni,xnj)|,

where *C*_*d*_(*i*, *j*) and *C*_*n*_(*i*, *j*) are defined as the absolute values of correlation coefficients between the expression profiles of genes *i* and *j* in the CML group and the normal group, respectively [18]; *x*_*di*_ and *x*_*dj*_ are the expression profiles of the *i*th and *j*th genes in the CML group; *x*_*ni*_ and *x*_*nj*_ are the expression profiles of the *i*th and* j*th genes in the normal group; cor(*x*_*di*_, *x*_*dj*_) and cor(*x*_*ni*_, *x*_*nj*_) are the Pearson correlation coefficients between them in the CML group and the normal group, respectively.

### 2.4. Classification of Coexpressed Gene Pairs

There was a set of correlation coefficients in either the normal group or the CML group. The two sets of correlation coefficients formed two cumulative distributions. We applied two-sample Kolmogorov-Smirnov (KS) test to identify the difference in the overall distributions of these two conditions (*C*_*d*_ and *C*_*n*_), including all the gene pairs. The maximum deviation between two cumulative distributions of *C*_*d*_ and *C*_*n*_ was identified (Formulas (2)), at which a threshold was found to classify the coexpressed gene pairs into strong and weak coexpression classes, called the disease-specific cutoff point (*C*). The cutoff point represented a coexpression level, at which *F*_*d*_ and *F*_*n*_ were extremely deviated. Gene pairs were further classified into four coexpression classes: (i) strongly coexpressed gene pairs in the normal group: pairs with coexpression levels (|*r*| values) bigger than or equal to *C* in the normal group; (ii) strongly coexpressed gene pairs in the CML group: pairs with coexpression levels (|*r*| values) bigger than or equal to *C* in the CML group; (iii) weakly coexpressed gene pairs in the normal group: pairs with coexpression levels (|*r*| values) smaller than *C* in the normal group; and (iv) weakly coexpressed gene pairs in the CML group: pairs with coexpression levels (|*r*| values) smaller than *C* in the CML group. Chi-square test was used to determine if the proportions of strongly and weakly coexpressed gene pairs significantly differed between the normal and the CML groups

(2) $$\begin{array}{c} D=\underset{C}{\max}\left|F_{d}\left(C\right)-F_{n}\left(C\right)\right|,\\ F_{d}\left(C\right)=\text{Prob}\left(C_{d}\geq C\right),\\ F_{n}\left(C\right)=\text{Prob}\left(C_{n}\geq C\right), \end{array}$$

where *F*_*d*_ and *F*_*n*_ are the cumulative distribution functions (CDFs) of *C*_*d*_ and *C*_*n*_, respectively; *D* is the maximum deviation; *C* is the cutoff point.

We further identified the specifically coexpressed gene pairs in different groups. Each type of gene pair represented a particular biological meaning. The normal-specific strongly coexpressed gene pairs were the gene pairs strongly coexpressed only in the normal group, which were regarded as the potential molecular interactions maintaining physiological balance in healthy individuals, and the impairment of these connections may lead to diseases. Obviously, these pairs were the CML-specific weakly coexpressed gene pairs, which were weakly coexpressed only in the CML group. The CML-specific strongly coexpressed gene pairs were the gene pairs strongly coexpressed only in the CML group, which represented the characteristics of the disease and may be the pathogenic alternatives when the corresponding normal-specific gene pairs cannot be coexpressed for responding to stress. Similarly, these pairs were regarded as the normal-specific weakly coexpressed gene pairs.

### 2.5. Functional Annotation for Candidate Target Genes

We applied* MetaCore* from GeneGo Inc. to annotate the candidate target genes. Specifically, when we uploaded the candidate target genes from Section 2.2 into this database, it mapped these genes to a set of cellular and molecular process networks, which are defined and annotated by Thomson Reuters scientists. In* MetaCore*, each process is defined as a preset network describing the protein interactions among them. In each process network, the annotated target genes were those genes included in both Section 2.2 and this process network. Enrichment analysis for a process network in* MetaCore* is performed based on the *P* value of hypergeometric intersection between the uploaded candidate target genes and the process-related genes in this database. The lower the *P* value is obtained, the higher the relevance of this process network to the candidate target genes and the rating of this process network are indicated. Only the top 10 statistically enriched process networks are shown according to the sorted *P* values in* MetaCore*.

### 2.6. Mapping Coexpressed Gene Pairs to Annotated Gene Pairs

The annotated target genes in each process network were paired with all the possible combinations to form the annotated gene pairs. The annotated gene pairs from each process network were mapped to the coexpressed gene pairs identified in Section 2.4: the normal-specific strongly coexpressed, the normal-specific weakly coexpressed, the CML-specific strongly coexpressed, and the CML-specific weakly coexpressed gene pairs. We applied Fisher's exact test to identify if there were more mapped normal-specific strongly coexpressed gene pairs than mapped CML-specific strongly coexpressed gene pairs in each process network. In other words, we planned to identify if these genes were more likely to be coexpressed in the normal group compared to the CML group. As a result, one-sided *P* value was chosen. False discovery rates (FDRs) are usually used to control the expected proportion of false positives for the multiple hypotheses. In this study, the FDRs were calculated based on the *P* values obtained from Fisher's exact test [19]. A process network was significantly mapped, if its FDR value was smaller than 0.05 [20]. The FDR values were estimated via the* Matlab* function,* mafdr* [21].

## 3. Results

### 3.1. Identification of Structural Coexpression Difference

**Figure 1:**
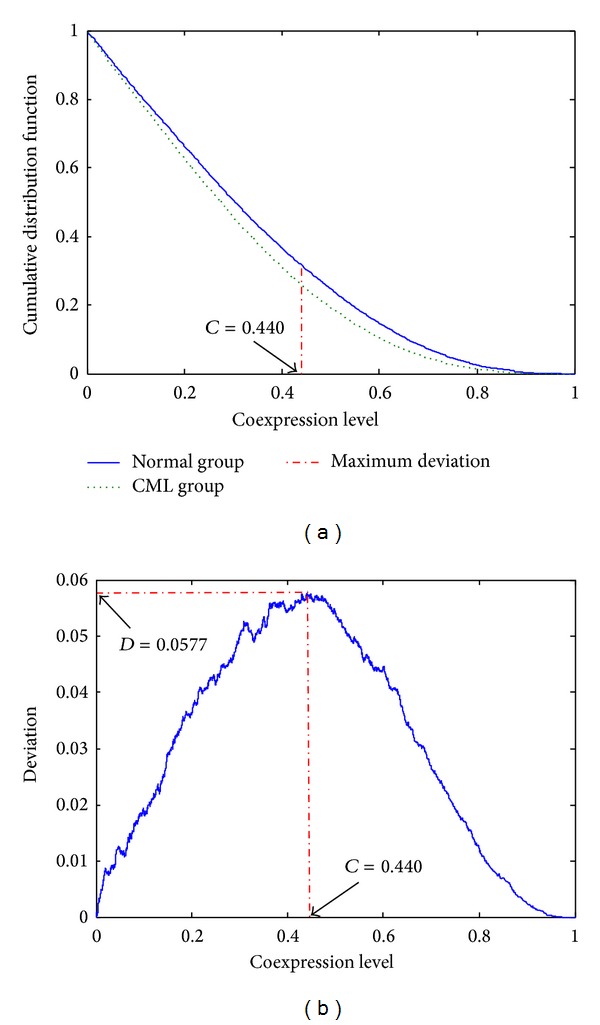
Plots of distributions for coexpression analysis. (a) Cumulative distribution functions of coexpression levels in the normal and the CML groups. (b) Deviation distribution against different coexpression cutoff points.

In total, we identified 217 common TGs of E2F1–3 and MYC that can be found in the microarray dataset GSE5550 (Table S1). We further extracted the available expression profiles of these TGs and calculated the correlation coefficients in both the normal and the CML groups. In each group, there was a set of correlation coefficients of 23,436 gene pairs. We plotted the cumulative distributions of these two sets of data. The distributions between the normal and the CML groups were significantly different (*P* value = 2.00 × 10^−34^ for *D* = 0.0577). The disease-specific cutoff point that classified the coexpressed gene pairs into strong and weak coexpression classes was *C* = 0.440 (Figure 1(a)).  Figure 1(b) illustrates that the deviation was small at the two extremes, and the peak (*D* = 0.0577) was found at the disease-specific cutoff point. Two coexpression patterns were so distinct that the normal group had more strongly coexpressed (level above ~0.440) and less weakly coexpressed (level below ~0.440) gene pairs than those in the CML group (Figure 1(a)). The cutoff point classified the gene pairs into four coexpression classes, shown in Table 1. The number of strongly coexpressed gene pairs in the normal group (7436) was larger than that in the CML group (6083). Chi-square test indicated that the proportions of strongly and weakly coexpressed gene pairs significantly differed between the normal and the CML groups (*P* value = 2.74 × 10^−43^ for *χ* ^2^ = 190).

### 3.2. *MetaCore* Analysis for Enriched Process Networks

The top 10 statistically enriched process networks for functional annotation of the 217 candidate target genes are shown in Table S2. All the *P* values for hypergeometric intersection test were smaller than 0.05. We got the annotated target genes involved in each process network and mapped the annotated gene pairs to the coexpressed gene pairs. Fisher's exact test was used to identify if there were more mapped normal-specific strongly coexpressed gene pairs than mapped CML-specific strongly coexpressed gene pairs in each process network. The results showed that 8 out of 10 process networks had more mapped normal-specific strongly coexpressed gene pairs (Table 2). Fisher's exact test demonstrated that “*Cell adhesion_Attractive and repulsive receptors*” and “*Development_Regulation of angiogenesis*” process networks were significantly mapped (*P* values = 0.001 and 0.012, <0.05, and FDR values were 0.004 and 0.026, <0.05).

**Figure 2:**
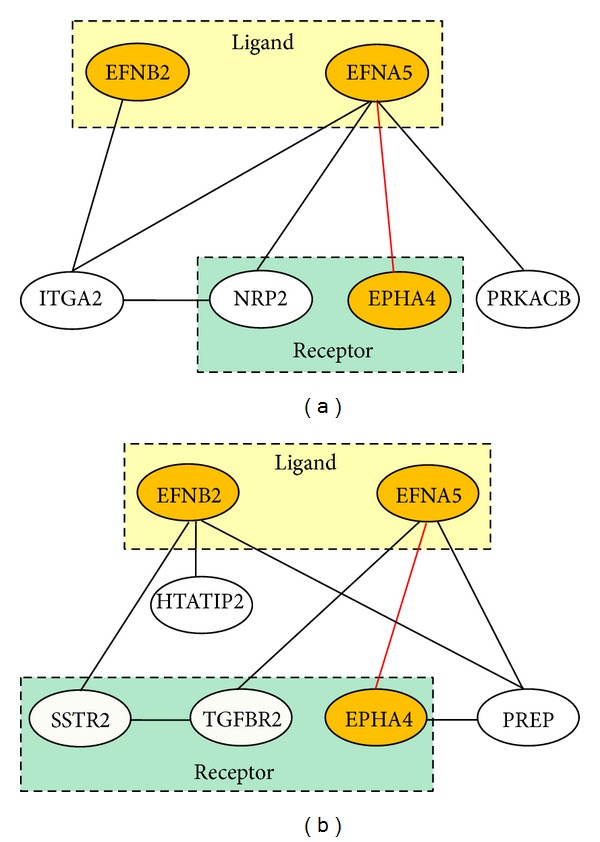
Coexpression networks for the mapped normal-specific strongly coexpressed gene pairs. The yellow ellipses are those genes found in both process networks. (a) Mapped normal-specific strongly coexpressed gene pairs in the “*Cell adhesion_Attractive and repulsive receptors*” process network. (b) Mapped normal-specific strongly coexpressed gene pairs in the “*Development_Regulation of angiogenesis*” process network.

We further plotted the coexpression networks for the mapped normal-specific strongly coexpressed gene pairs (*a *= 6 and 8) (Figure 2). Both “*Cell adhesion_Attractive and repulsive receptors*” and “*Development_Regulation of angiogenesis*” process networks had ephrin-B2 (EFNB2), ephrin-A5 (EFNA5), and EPH receptor A4 (EPHA4) (Figure S2). From* National Center for Biotechnology Information *(*NCBI*) database, we obtained the basic information for these genes/proteins. EFNB2 and EFNA5 are the members of the ephrin gene family. EPHA4 protein product is an ephrin receptor. The ephrins (EPH) and EPH-related receptors belong to the largest subfamily of receptor protein-tyrosine kinases, which play a vital role in mediating developmental events. Figure 2 shows that the connection from EFNA5 to EPHA4 was identified as a strongly coexpressed gene pair for these two process networks in the normal group. In addition, protein products from neuropilin 2 (NRP2), transforming growth factor, beta receptor II (TGFBR2), and somatostatin receptor 2 (SSTR2) also belong to receptors, which are very important in signal transduction process. The encoded protein from integrin, alpha 2 (ITGA2), plays a vital role in leukocyte intercellular adhesion process. There were three enzymes identified in the coexpression networks: (i) the protein encoded by protein kinase, cAMP-dependent, catalytic, beta (PRKACB) is a protein kinase; (ii) the protein product from prolyl endopeptidase (PREP) is a protease; and (iii) the protein encoded by HIV-1 Tat interactive protein 2 (HTATIP2) is an oxidoreductase required for tumor suppression. From the results, we can infer that these genes/proteins were well connected with each other to transduce signals and maintain physiological balance in healthy individuals. However, in the CML group, these connections were impaired.

## 4. Discussion and Conclusion

In this study, our developed method successfully identified the difference in the coexpression patterns of those candidate target genes regulated concurrently by E2F1–3 and MYC between the normal and the CML groups from the overall structure (Figure 1). We further found that genes involved in the cell adhesion and angiogenesis properties were more likely to be coexpressed in the normal group compared to the CML group (Table 2 and Figure 2). The alteration in adhesion properties of leukemic progenitors is one CML characteristic at the cellular level [1]. In addition, Bhatia et al. hypothesized that decreased integrin-mediated adhesion of CML progenitors to stroma can lead to continuous cell proliferation [22]. They treated the cells with interferon-*α* (IFN-*α*). The results showed that the treatment restored the CML progenitor adhesion to stroma and also the regulation of CML progenitor proliferation [22]. Angiogenesis is the process forming new blood from the preexisting vasculature, including degradation of extracellular matrix proteins, as well as activation, proliferation, and migration of endothelial cells [23]. In leukemia, hematopoietic cells are supported from the normal vascular bed in bone marrow [23]. Increased vascularity was found in acute myeloid leukemia (AML) patients [24]. Importantly, in CML, the number of blood vessels and vascular areas were found to be increased when compared to control bone marrows [23]. Our results showed that the connection from EFNA5 to EPHA4 was identified as a strongly coexpressed gene pair in the normal group (|*r*|  values were 0.720 and 0.013 in the normal group and the CML group, resp.) (Figure 2). Ephrin-A receptors belong to the largest subfamily of receptor tyrosine kinases that regulate cell shape, mobility, and attachment [25]. Interactions between Ephrin-A receptors and ligands are important in cell-cell communication, initiating unique bidirectional signaling cascades to transduce the information [26]. There may be some relationships between adhesion property and angiogenesis. These two process networks were found to be well controlled in the normal group compared to the CML group. Dysregulation of adhesion and angiogenesis properties is a possible reason leading to CML.

The advantage of our study is the application of coexpression analysis to target genes regulated concurrently by more than one transcription factor under different conditions. We identified different coexpression patterns between the normal and the CML groups. A limitation for differential expression analysis is that it only reflects the upregulation or downregulation of existing components in the well-known pathways under the normal or the disease condition, which cannot identify the functionally associated linkages among genes during signal transduction. In addition, differential expression analysis does not take account of the level of correlations that may exist between gene expression patterns [12]. Coexpression analysis is useful for analyzing the underlying mechanisms of diseases. Moreover, the different coexpression pattern can be regarded as the signature of a disease.

Several methods have been proposed to analyze coexpressed genes. The two-stage screening procedure was applied to select statistically and biologically significant gene pairs in Zhu et al.'s study [27]. Gupta et al. proposed a method for determining the correlation threshold using the clustering coefficient. *R* ^2^ metric was used as a measure of similarity between two genes [28]. Previous studies cannot reflect the overall difference between two different groups. Our method calculated all the correlation coefficients in each group (the normal group and the CML group) to form two distributions, which can find the difference between two different groups from the overall structure.

In summary, we have presented a detailed method to identify a disease-specific cutoff point for coexpression levels that classified the coexpressed gene pairs into strong and weak coexpression classes so that the class was best coherent with the disease phenotype. We applied this method to explore the difference in the coexpression patterns of target genes regulated concurrently by E2F1–3 and MYC between the normal and the CML groups. Our method effectively identified the statistical differences between the normal and the CML groups from the overall structure. We further found the potentially altered cell adhesion and angiogenesis properties in the CML state when compared to the normal group. The different coexpression pattern can reflect the biological alterations in CML. Our significant findings will be helpful in exploring the underlying mechanisms of CML and provide useful information in cancer treatment.

## Supplementary Material

The flow chart describing the E2F1–3 and MYC target genes co-expression analysis is shown in Figure S1. The functional annotation from MetaCore for the candidate target genes involved in “Cell adhesion_Attractive and repulsive receptors” and “Development_Regulation of angiogenesis” process networks is shown in Figure S2. The 217 candidate target genes of E2F1–3 and MYC that can be found in the microarray dataset GSE5550 are shown in Table S1. The top 10 statistically enriched process networks from MetaCore for the functional annotation of the 217 candidate target genes are shown in Table S2.

## Figures and Tables

**Table 1:** The coexpressed gene pairs identified by the disease-specific cutoff point.

Group	Number of strongly coexpressed pairs	Number of weakly coexpressed pairs
Normal	7436	16000
CML	6083	17353

**Table 2:** Mapping coexpressed gene pairs to annotated gene pairs from each process network.

Process networks	Fisher's exact test					FDR
	*a*	*b*	*c*	*d*	*P* value	
Development_Neurogenesis in general	14	11	11	14	0.286	0.251
Development_Hedgehog signaling	22	15	15	22	0.081	0.118
Signal transduction_WNT signaling	10	7	7	10	0.247	0.270
Signal transduction_TGF-beta, GDF, and activin signaling	6	5	5	6	0.500	0.365
Cell adhesion_Attractive and repulsive receptors	**6**	**0**	**0**	**6**	***0.001***	***0.004***
Development_Regulation of angiogenesis	**8**	**2**	**2**	**8**	***0.012***	***0.026***
Cardiac development_BMP_TGF_beta_signaling	2	1	1	2	0.500	0.313
Neurophysiological process_Melatonin signaling	3	2	2	3	0.500	0.274

*a*: mapped normal-specific strongly coexpressed gene pairs; *b*: mapped normal-specific weakly coexpressed gene pairs; *c*: mapped CML-specific strongly coexpressed gene pairs; *d*: mapped CML-specific weakly coexpressed gene pairs.
