# Supplementary material for: Novel Approach for Coexpression Analysis of E2F1–3 and MYC Target Genes in Chronic Myelogenous Leukemia
Source: Biomed Res Int. 2014 Aug 10;2014:439840. doi: 10.1155/2014/439840 (PMC4142389; doi:10.1155/2014/439840)
Supplement: Supplementary file 1 — The flow chart describing the E2F1–3 and MYC target genes co-expression analysis is shown in Figure S1. The functional annotation from MetaCore for the candidate target genes involved in “Cell adhesion_Attractive and repulsive receptors” and “Development_Regulation of angiogenesis” process networks is shown in Figure S2. The 217 candidate target genes of E2F1–3 and MYC that can be found in the microarray dataset GSE5550 are shown in Table S1. The top 10 statistically enriched process networks from MetaCore for the functional annotation of the 217 candidate target genes are shown in Table S2. [file 439840.f1.zip › 439840.f1/Figure S2.pdf]

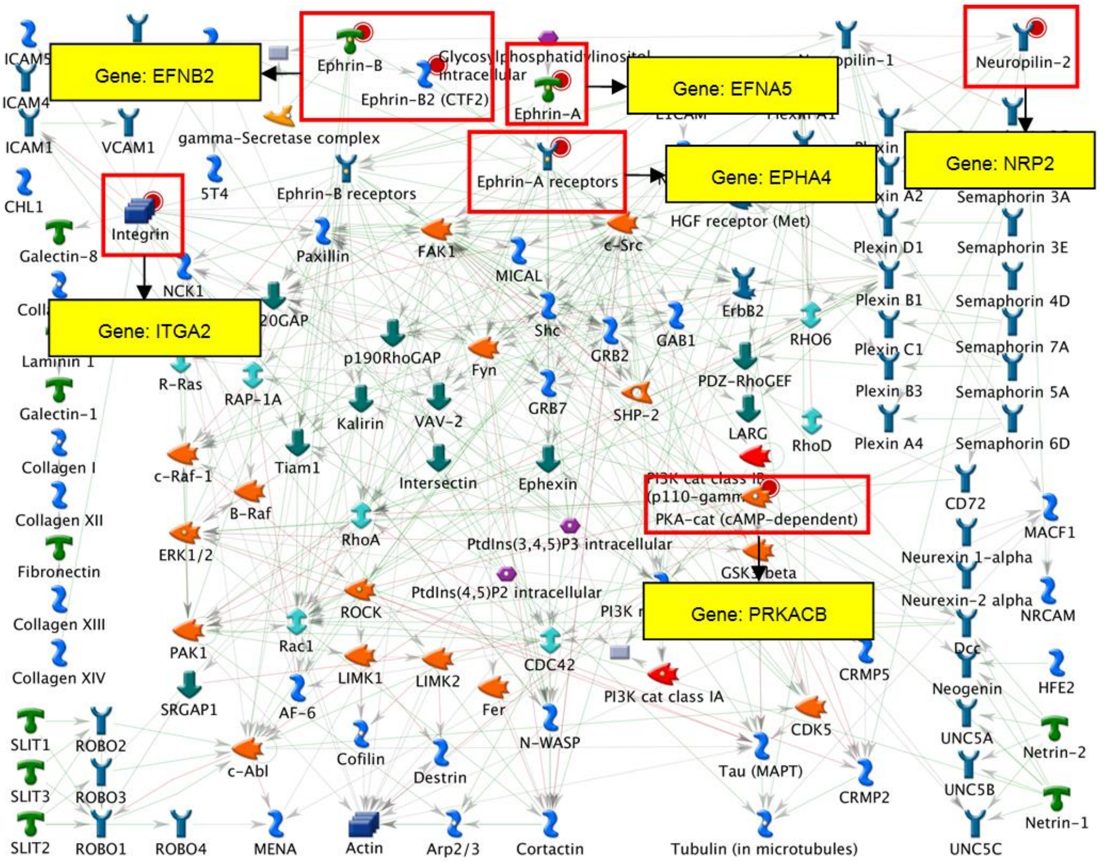

Gene: EFNB2
Gene: EFNA5
Gene: EPHA4
Gene: NRP2
Gene: ITGA2
Gene: PRKACB
ICAM4
ICAM1
VCAM1
CHL1
Integrin
Galectin-8
Ephrin-B
Ephrin-B2 (CTF2)
gamma-Secretase complex
5T4
Ephrin-B receptors
Ephrin-A
Ephrin-A receptors
Neuropilin-2
HGF receptor (Met)
Semaphorin 3A
Semaphorin 3E
Semaphorin 4D
Semaphorin 7A
Semaphorin 5A
Semaphorin 6D
Plexin A2
Plexin D1
Plexin B1
Plexin C1
Plexin B3
Plexin A4
NCK1
Paxillin
FAK1
c-Src
MICAL
ErbB2
Shc
GRB2
GAB1
RHO6
p190RhoGAP
Fyn
PDZ-RhoGEF
Laminin 1
R-Ras
RAP-1A
Galectin-1
Kalirin
VAV-2
GRB7
SHP-2
LARG
RhoD
Collagen I
c-Raf-1
Tiam1
Intersectin
Ephexin
B-Raf
Collagen XII
PKA-cat (cAMP-dependent)
CD72
MACF1
RhoA
PtdIns(3,4,5)P3 intracellular
ERK1/2
Fibronectin
Neurexin 1-alpha
Collagen XIII
PtdIns(4,5)P2 intracellular
Neurexin-2 alpha
NRCAM
ROCK
PAK1
Rac1
CDC42
CRMP5
Collagen XIV
LIMK1
LIMK2
Fer
PI3K cat class IA
Neogenin
HFE2
SRGAP1
AF-6
N-WASP
CDK5
UNC5A
SLIT1
ROBO2
c-Abl
Cofilin
Destrin
Tau (MAPT)
Netrin-2
SLIT3
ROBO3
CRMP2
UNC5B
Netrin-1
SLIT2
ROBO1
ROBO4
MENA
Actin
Arp2/3
Cortactin
Tubulin (in microtubules)
UNC5C


(a)

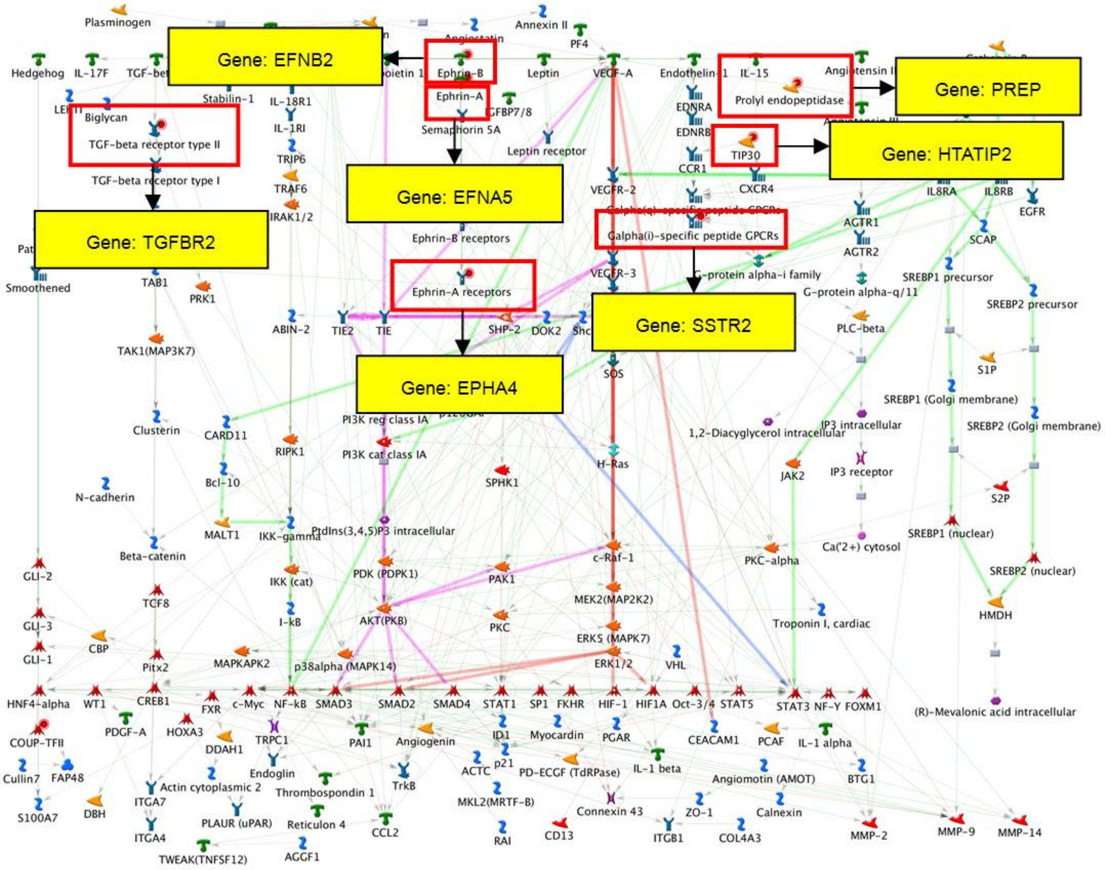


(b)

Figure S2. Functional annotation for the candidate target genes in *MetaCore* process networks. The red rectangles refer to those genes found in the mapped normal-specific strongly co-expressed gene pairs. (a) "*Cell adhesion_Attractive and repulsive receptors*" process network. (b) "*Development_Regulation of angiogenesis*" process network.
