# Supplementary material for: Novel Approach for Coexpression Analysis of E2F1–3 and MYC Target Genes in Chronic Myelogenous Leukemia
Source: Biomed Res Int. 2014 Aug 10;2014:439840. doi: 10.1155/2014/439840 (PMC4142389; doi:10.1155/2014/439840)
Supplement: Supplementary file 1 — The flow chart describing the E2F1–3 and MYC target genes co-expression analysis is shown in Figure S1. The functional annotation from MetaCore for the candidate target genes involved in “Cell adhesion_Attractive and repulsive receptors” and “Development_Regulation of angiogenesis” process networks is shown in Figure S2. The 217 candidate target genes of E2F1–3 and MYC that can be found in the microarray dataset GSE5550 are shown in Table S1. The top 10 statistically enriched process networks from MetaCore for the functional annotation of the 217 candidate target genes are shown in Table S2. [file 439840.f1.zip › 439840.f1/Table S2.pdf]

Table S2. The top 10 enriched process networks from *MetaCore* for functional annotation of 217 candidate target genes.

#	Significant process networks	Total genes	In our data	p-value
1	Cell adhesion_Synaptic contact	184	13	1.467×10^{-4}
2	Development_Neurogenesis in general	192	12	8.064×10^{-4}
3	Development_Hedgehog signaling	254	14	1.038×10^{-3}
4	Signal transduction_WNT signaling	177	11	1.394×10^{-3}
5	Signal Transduction_TGF-beta, GDF and Activin signaling	154	10	1.654×10^{-3}
6	Cell adhesion_Attractive and repulsive receptors	175	10	4.204×10^{-3}
7	Reproduction_FSH-beta signaling pathway	160	9	7.211×10^{-3}
8	Development_Regulation of angiogenesis	223	11	8.168×10^{-3}
9	Cardiac development_BMP_TGF_beta_signaling	117	7	1.252×10^{-2}
10	Neurophysiological process_Melatonin signaling	43	4	1.304×10^{-2}
