# Supplementary figures and images for: Novel Approach for Coexpression Analysis of E2F1–3 and MYC Target Genes in Chronic Myelogenous Leukemia
Source: Biomed Res Int. 2014 Aug 10;2014:439840. doi: 10.1155/2014/439840 (PMC4142389; doi:10.1155/2014/439840)

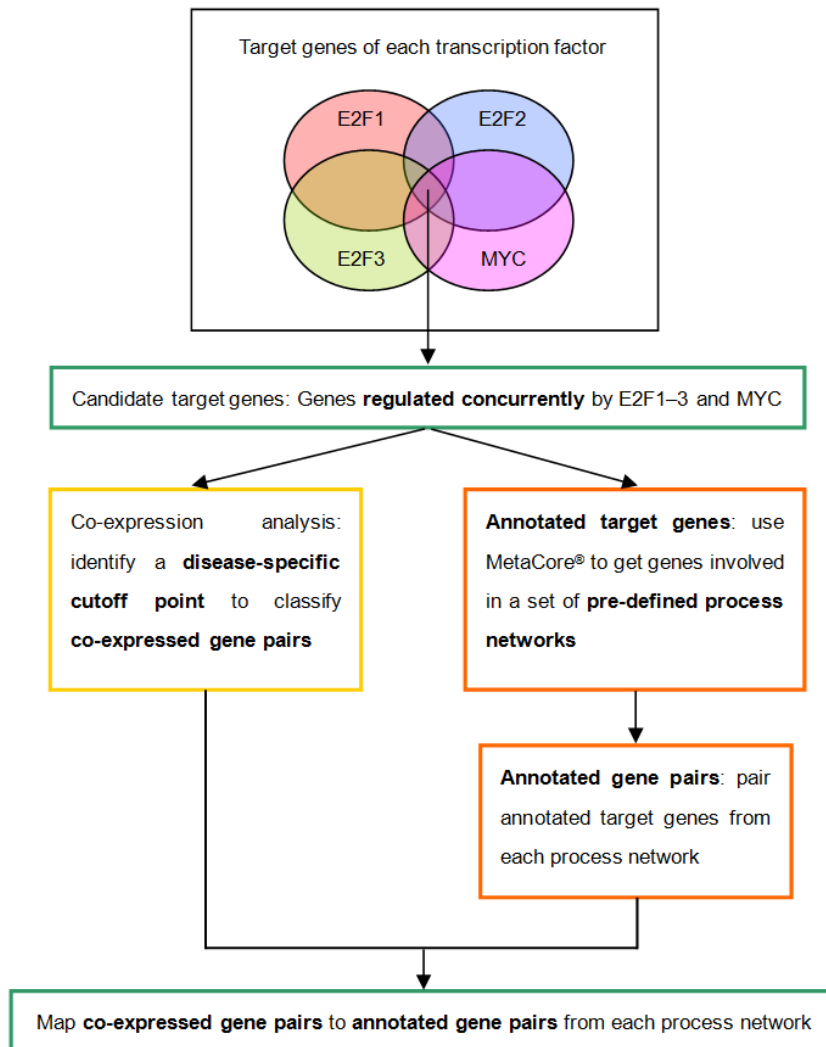

Figure S1. Flow chart for the E2F1-3 and MYC target genes co-expression analysis.

Supplement: Supplementary file 1 — The flow chart describing the E2F1–3 and MYC target genes co-expression analysis is shown in Figure S1. The functional annotation from MetaCore for the candidate target genes involved in “Cell adhesion_Attractive and repulsive receptors” and “Development_Regulation of angiogenesis” process networks is shown in Figure S2. The 217 candidate target genes of E2F1–3 and MYC that can be found in the microarray dataset GSE5550 are shown in Table S1. The top 10 statistically enriched process networks from MetaCore for the functional annotation of the 217 candidate target genes are shown in Table S2. [file 439840.f1.zip › 439840.f1/Figure S1.pdf]
